# Early Recognition of Burn- and Trauma-Related Acute Kidney Injury: A Pilot Comparison of Machine Learning Techniques

**DOI:** 10.1038/s41598-019-57083-6

**Published:** 2020-01-14

**Authors:** Hooman H. Rashidi, Soman Sen, Tina L. Palmieri, Thomas Blackmon, Jeffery Wajda, Nam K. Tran

**Affiliations:** 1 Department of Pathology and Laboratory Medicine, 4400 V Street, Sacramento, CA 95817 USA; 2 Division of Burn Surgery, Department of Surgery, 2221 Stockton Blvd., Sacramento, CA 95817 USA; 3 UC Davis Health (2315 Stockton Blvd, Sacramento, CA 95817 USA

**Keywords:** Diagnostic markers, Laboratory techniques and procedures

## Abstract

Severely burned and non-burned trauma patients are at risk for acute kidney injury (AKI). The study objective was to assess the theoretical performance of artificial intelligence (AI)/machine learning (ML) algorithms to augment AKI recognition using the novel biomarker, neutrophil gelatinase associated lipocalin (NGAL), combined with contemporary biomarkers such as N-terminal pro B-type natriuretic peptide (NT-proBNP), urine output (UOP), and plasma creatinine. Machine learning approaches including logistic regression (LR), *k*-nearest neighbor (*k*-NN), support vector machine (SVM), random forest (RF), and deep neural networks (DNN) were used in this study. The AI/ML algorithm helped predict AKI 61.8 (32.5) hours faster than the Kidney Disease and Improving Global Disease Outcomes (KDIGO) criteria for burn and non-burned trauma patients. NGAL was analytically superior to traditional AKI biomarkers such as creatinine and UOP. With ML, the AKI predictive capability of NGAL was further enhanced when combined with NT-proBNP or creatinine. The use of AI/ML could be employed with NGAL to accelerate detection of AKI in at-risk burn and non-burned trauma patients.

## Introduction

Acute kidney injury (AKI) is a common complication among critically ill patients^1–4^. Severely burned patients, in particular, have been shown to be at high-risk with up to 58% experiencing AKI^3–5^. The early recognition of AKI helps guide fluid resuscitation and titrate dosing of nephrotoxic drugs in these populations. Unfortunately, traditional biomarkers of renal function such as creatinine and urine output (UOP) have been shown to be suboptimal at predicting AKI^6,7^. Novel AKI biomarkers have been proposed, but widespread use in the United States remains limited.

Advances in computational technology have rapidly facilitated the growth of artificial intelligence (AI) and machine learning (ML)^8,9^. Studies have reported AI/ML aiding in the diagnosis of several disease and perhaps augment the performance of existing tests with varying degrees of success^10–13^. Interestingly, recent investigations postulated AI/ML using a *k*-nearest neighbor (*k*-NN) approach could augment the identification of AKI in burn patients using only plasma creatinine, UOP and *N*-terminal pro-B-type natriuretic peptide (NT-proBNP)^14^. Notably, that study was limited to burn patients—raising the question if these algorithms could apply to other critically ill populations and if *k*-NN was the optimal ML technique for AKI prediction.

Severely burned patients have been shown to be fundamentally different from traditional non-burned trauma populations^15,16^. Interestingly, AKI classification remains the same between both populations and based on the Kidney Disease and Improving Global Outcomes (KDIGO) criteria^17^. This similarity offers a unique opportunity to determine if ML models developed in one population (*i.e*., burn patients) could be translated to another (*i.e*., non-burned trauma patients) and how KDIGO performs against such ML techniques. Notably, the KDIGO criteria relies solely on UOP and creatinine measurements which has shown poor performance in burn patients, therefore combining ML with other biomarkers of AKI and cardiorenal syndrome may have clinical merit. *To this end, the goal of this study was to determine if a burn-trained ML algorithm could be generalized to a non-burned population and evaluate the value of including novel renal injury biomarker combinations to enhance AKI prediction*.

## Methods

We developed, validated, and compared five ML algorithms for early recognition of AKI following Cross Industry Standard Process for Data Mining (CRISP-DM) guidelines for a combined population of burn and non-burned trauma surgery patients. Selected features were NGAL, creatinine, NT-proBNP, and UOP based on their significance and relevance in clinical practice. The study focused on ML prediction within the first 24 hours due to burn- and/or trauma injury-related shock being common mechanisms causing AKI. These algorithms were first trained and validated on a retrospective burn AKI dataset. We then determined the generalizability of these ML algorithms in a second dataset containing a mix of burned and non-burned trauma surgery patients. The study was approved by the University of California, Davis Institutional Review Board (Study Cohort A: Protocol# 214836, and Study Cohort B: Protocol#1085450). All methods were performed in accordance with the relevant guidelines and regulations. Informed consent was obtained for all subjects.

### Retrospective burn study population (Cohort A)

The retrospective quality database consisted of 50 adult (age ≥18 years) patients with ≥20% total body surface area (TBSA) burns at risk for AKI reported previously^14^. This database was derived from a hospital clinical laboratory project to validate a commercially available plasma neutrophil gelatinase associated lipocalin (NGAL) enzyme linked immunosorbent assay (Bioporto, Inc, Denmark). NGAL testing was performed on residual plasma chemistry samples collected at the time of burn intensive care unit admission. Briefly, NGAL is a novel AKI biomarker and is released by neutrophils during inflammation and renally cleared^6,7^. During AKI, decreases in glomerular filtration rate (GFR) increases plasma concentrations of NGAL. Unique to NGAL, renal tubular cells also produce the biomarker during AKI—increasing both plasma and urine concentrations of NGAL.

In addition to NGAL, we included natriuretic peptide testing given AKI can lead to acute heart dysfunction and manifesting as cardiorenal syndrome^6,7,18^. Specifically, *N*-terminal pro B-type natriuretic peptide (NT-proBNP) was also measured (Roche Diagnostics, Indianapolis, IN) using the same plasma samples. Paired to the NGAL and NT-proBNP results, we also recorded UOP, plasma creatinine results, and vital signs from the electronic medical record (EMR). Chart review was used to determine which patients experienced AKI during the first one-week of burn intensive care unit admission based on KDIGO criteria.

### Prospective burn and trauma population (Cohort B)

The second dataset consisted of 51 adult patients with ≥20% TBSA burns or non-burn trauma-related injuries requiring surgery. Inclusion of a non-burned trauma population served to determine the generalizability of each ML model. These patients were prospectively enrolled to obtain residual clinical plasma samples within the first 24 hours of admission for testing by the same NGAL and NT-proBNP assays to predict AKI. Both NGAL and NT-proBNP results were not used for patient care. Again, chart review was performed to obtain paired UOP and plasma creatinine results, as well as patient history, vital signs (*i.e*., mean arterial pressure, central venous pressure) and demographic data. KDIGO criteria^17^ was used to determine AKI status within the first week of stay.

### ML algorithms

**Figure 1:**
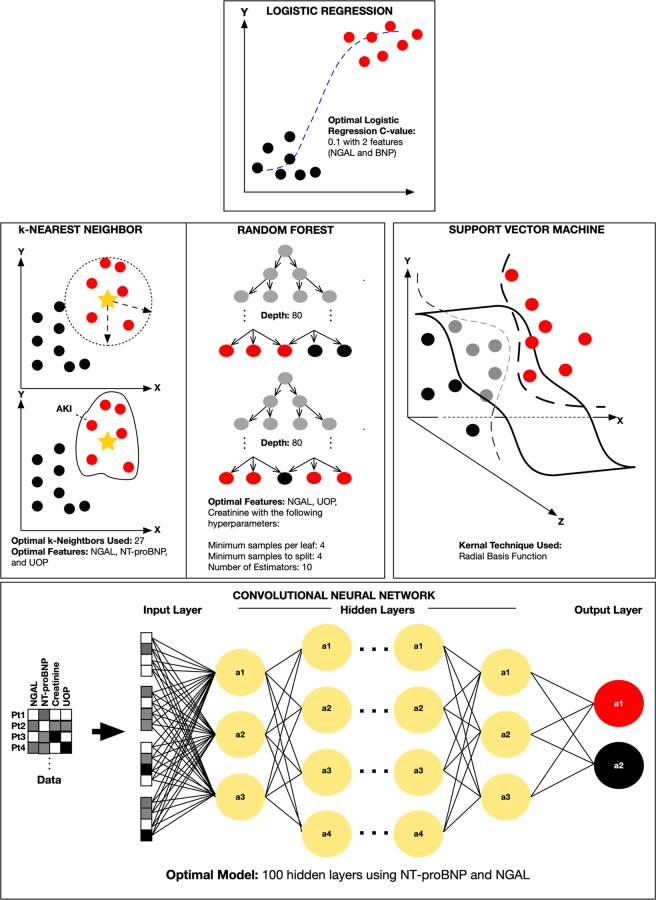
Comparison of DNN, LR, *k*-NN, RF, and SVM: The figure compares the five ML techniques used in the study and illustrated as conceptual drawings with optimal parameters used in the study serving as examples. Red circles indicate acute kidney injury (AKI) patients, black circles indicate non-AKI patients, and grey circles indicate unclassified patients. At the top, is LR. Middle row from left to right is *k*-NN, RF, and SVM respectively. The bottom row illustrates a DNN where each patient (Pt#) data matrix containing various combinations of biomarkers and their respective levels (white: none, grey: low, black: high) are processed by hidden layers for classification as having AKI or no AKI.

Five ML approaches were evaluated to differentiate AKI versus non-AKI patients (Fig. 1). Cohort A was used for the initial training and testing. This was then followed by Cohort B serving as means to evaluate the overall generalizability of our best performing ML algorithms. These ML approaches included: (a) logistic regression (LR), (b) *k*-nearest neighbor (*k*-NN), (c) random forest (RF), (d) support vector machine (SVM), and our multi-layer perceptron (MLP) deep neural network (DNN) (Fig. 1). The Scikit-Learn’s version 0.20.2 was used in constructing the models within all five algorithms. Briefly, LR is based on traditional statistical techniques that is generally used for identifying predictors of a binary outcome (*i.e*., AKI vs. no AKI). *k*-NN is a non-parametric pattern recognition algorithm used for classification and regression^19^. Classification is based on the number of *k* neighbors and typically its Euclidean distance (*d*) from a pre-defined point. In contrast, random forest, a form of ensemble learning, uses a multitude of constructed decision trees for classification and regression^20^. Next, SVM is a form of AI/ML that classifies data by defining a hyperplane that best differentiates two groups (*i.e*., AKI vs. non-AKI patients) by maximizing the margin (the distance), ultimately leading to a hyperplane-bounded region with the largest possible margin^21^. Thus, the goal of SVM is to maximize the distance (margin) between groups of data which can also be applied as a linear method to nonlinear data by transposing the data features into a higher dimension (*e.g*., three dimensions) through the use of kernels. For this study, our SVM model incorporated a radial basis function kernel technique. This ultimately allows for a better classification and differentiation of the groups of interest (*e.g*., AKI versus No-AKI). Lastly, DNN utilizes artificial neural networks with multiple levels between input and output layers. Ultimately these multi-layer perceptrons (MLP) within the DNN identifies the appropriate mathematical manipulation to convert an input into an output. Our custom multi-layer neural network grid search in the scikit learn library uses the “Adam” solver (a stochastic gradient-based optimizer) to generate our multi-layer neural networks. This along with our variable number of hidden layers, variable penalty regularization alpha parameters, variable tol values (tolerance for the optimization parameters) and two unique activation functions: ReLU (the rectified linear unit function) and tanh (hyperbolic tan function) allowed us to build and find our best performing multi-layer neural network for each category amongst the thousands of our uniquely constructed ML models^22,23^. Since these ML algorithms are sensitive to unscaled data, variables were scaled based on a standard scaler method transforming features to a mean of 0 with a standard deviation of 1^14^.

### Cross validation studies

Cross validation studies were also performed for LR, RF, *k*-NN, SVM, and DNN methods using the Scikit-learn cross validation grid search tool. This technique along with the grid search hyperparameter variations (noted above) enabled us to build and compare unique models to yield a total of 68,100 ML models within our five ML methods/algorithms and our unique categories. Using this approach, we were able to empirically assess and compare the performance of all these models which ultimately lead to identifying the best performing ML models with a unique set of hyperparameters within each ML method and feature set combinations. The mean accuracy for each set of these models were then analyzed.

### Statistical analysis

JMP software (SAS Institute, Cary, NC) was used for statistical analysis. Describe statistics were calculated for patient demographics. The Shapiro-Wilkes test and histogram analysis were used to determine normality. Continuous normally distributed variables were compared using means (standard deviation [SD]) using the 2-sample *t*-test, while discrete variables were compared using the non-parametric Chi-square test. Non-parametric continuous data compared using medians (interquartile range [IQR]), when appropriate, were analyzed using the Mann-Whitney U test. Multivariate logistic regression was used to determine predictors of AKI with age and burn size serving as covariates. Repeated measures analysis of variance was used for time series data. A *p*-value < 0.05 was considered statistically significant with receiver operator characteristic (ROC) analysis also performed to compare AKI biomarker performance.

## Results

Patient demographics and biomarker comparisons between study cohorts (A vs. B, AKI vs. non-AKI, and burned vs. non-burned groups) are shown in Table 1. Briefly, 50% of patients (25/50) in Cohort A experienced AKI within the first week of hospital stay as shown previously. Five patients experienced fluid overload manifested as compartment syndrome. Again, Cohort A served as the dataset for our training phase and initial validation testing. In contrast, in our Cohort B 21.6% (11/51) of the patients experienced AKI within the same timeframe. Eight patients experienced over-resuscitation presenting with compartment syndrome (n = 2), pulmonary edema (n = 2), or both compartment syndrome and pulmonary edema (n = 4). Leveraging both some population similarities and differences, Cohort B was used as our secondary testing dataset to assess the generalizability of the models generated from cohort A. The mean (standard deviation [SD]) time for patients to meet KDIGO AKI criteria was 42.7 (23.2) hours for Cohort A and 71.5 (39.5) hours for Cohort B.

**Table 1:** Patient Demographics and Comparison of Biomarker Levels (Page 1 of 2).

COHORT A - TRAINING	Burn AKI	Burn Non-AKI		
	(n = 25)	(n = 25)		
Mean (SD) Age (years)	39.1 (49.2)	39.7 (15.5)		
Gender (M/F)	20/5	19/6		
Burn Size (%TBSA)	49.2 (24.1)	43.3 (18.9)		
Mean (SD) Arterial Pressure (mmHg)	78.9 (11.5)	80.1 (5.2)		
Mean (SD) Central Venous Pressure (mmHg)	13.3 (3.4)	12.0 (7.6)		
Mean (SD) Creatinine (mg/dL)	1.21 (0.51)	0.90 (0.22)		
Mean (SD) NGAL (ng/mL)	185.1 (86.3)	110.3 (48.1)		
Mean (SD) NT-proBNP (pg/mL)	25.7 (15.4)	16.0 (15.3)		
Mean (SD) UOP (mL/hr)	81.5 (31.6)	85.7 (48.9)		
Mean (SD) Time to AKI (hours)^a^	42.7 (23.2)	N/A		
**COHORT B - TEST**	**Burn AKI**	**Burn Non-AKI**	**Trauma AKI**	**Trauma Non-AKI**
	**(n = 6)**	**(n = 15)**	**(n = 7)**	**(n = 23)**
Mean (SD) Age (years)	38.2 (41.5)	40.1 (20.2)	37.6 (39.9)	39.1 (19.5)
Gender (M/F)	4/2	12/3	4/3	15/10
Burn Size (%TBSA)	41.1 (14.8)	40.0 (20.4)	N/A	N/A
Mean (SD) Arterial Pressure (mmHg)	82.8 (15.5)	79.7 (18.3)	70.3 (20.8)	75.1 (20.3)
Mean (SD) Central Venous Pressure (mmHg)	12.6 (4.4)	12.9 (5.8)	10.7 (6.2)	12.3 (6.9)
Mean (SD) Creatinine (mg/dL)	2.15 (1.77)	0.93 (0.46)	2.16 (1.57)	0.86 (0.32)
Mean (SD) NGAL (ng/mL)	300.4 (213.5)	110.0 (39.7)	396.7 (393.7)	77.4 (32.1)
Mean (SD) NT-proBNP (pg/mL)	144.3 (23.6)	57.5 (16.9)	137.3 (62.1)	93.7 (10.4)
Mean (SD) UOP (mL/hr)	47.7 (41.2)	93.3 (41.1)	66.1 (37.2)	87.4 (58.2)
Mean (SD) Time to AKI (hours)^a^	43.9 (15.3)	N/A	82.7 (38.6)	N/A

Note: ^a^Time from admission to achieving AKI based on KDIGO criteria.

Abbreviations: F, female; KDIGO, Kidney Disease: Improving Global Outcomes; M, male; mmHg, millimeters mercury; mL, milliliter; ng, nanogram; NGAL, neutrophil gelatinase associated lipocalin; N/A, not applicable; NT-proBNP; N-terminal pro-B-type natriuretic peptide; pg, picogram; SD, standard deviation; TBSA, total body surface area; and UOP, urine output.

Focusing on Cohort B, which was our “secondary test/generalizability” population, median (IQR) plasma creatinine (1.17 [1.52] vs. 0.83 [0.53], P < 0.001) and UOP (66.4 [79.3] vs. 86.5 [53.6] mL/hour, P = 0.023) statistically different between AKI versus non-AKI groups. Mean NT-proBNP was significantly higher in the AKI group (107.0 [53.3] vs. 60.4 [13.2] pg/mL, P = 0.016). NGAL served as an independent predictor of AKI (OR 2.7, 95% CI 0.8–4.5, P < 0.001) and concentrations were found to be significantly higher among the AKI patients (260.7 [163.8] vs. 89.6 [38.1] ng/mL, P = 0.006). However, there were no statistically significant differences between burned vs. non-burned AKI patients for mean plasma creatinine (2.15 [1.77] vs. 2.16 [1.58] mg/dL, P = 0.984), UOP (47.8 [41.2] vs. 66.1 [37.2] mL/hour, P = 0.422), and mean NT-proBNP (114.3 [23.6] vs. 137.3 [93.7] pg/mL, P = 0.551). The average time from admission to meeting KDIGO AKI criteria was significantly different between burned versus non-burned patients respectively (43.9 [15.3] vs. 82.7 [38.6], P = 0.029).

Comparing non-AKI patients with burn injury versus those without, mean NGAL concentrations were significantly higher among the non-burned population (109.9 [39.7] vs. 77.4 [32.1] ng/mL, P = 0.013), while mean NGAL levels between burned versus non-burned AKI patients were similar (300.4 [213.5] vs. 396.7 [393.7] ng/mL, P = 0.589). Sub-group analysis among Cohort A and B burn patients experiencing fluid overload complications (*i.e*., compartment syndrome and/or pulmonary edema) showed significantly high mean NT-proBNP levels (Cohort A [n = 5]: 78.2 [15.8] pg/mL vs. Cohort B [n = 8], 372.4 [10.7] pg/mL, P < 0.001).

Receiver operator characteristics analysis showed NGAL serving as the best AKI biomarker (area under the curve [AUC]: 0.93, P = 0.023), followed by NT-proBNP (0.85), plasma creatinine (0.68), and UOP (0.57). The area under the ROC curve for each biomarker was significantly (P = 0.038) larger among non-burned patients versus burned patients.

### ML modeling and comparisons with Cohort B

**Figure 2:**
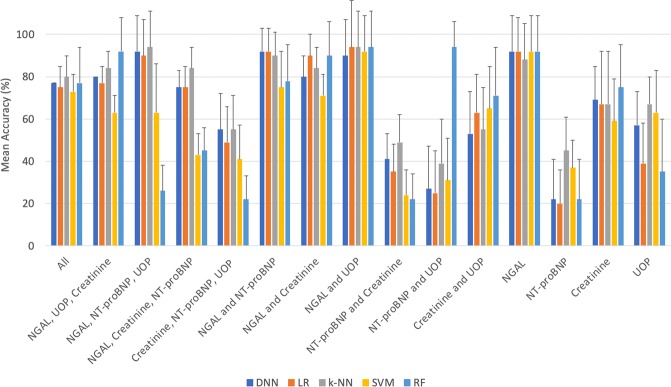
Accuracy of Cohort B Data Used for Generalization oF DNN, LR, *k*-NN, SVM and RF Algorithms: Bar graphs illustrate the accuracy for each of the five AI/ML techniques with differing combinations of NGAL, UOP, plasma creatinine, and NT-proBNP. Data was based on Cohort B (n = 51) severely burned or non-burned trauma patients. Notably, the accuracy and sensitivity of best performing models with NGAL alone was 92% and 73%, with an AUC of 85 respectively in 4 out of the five algorithms while the accuracy and sensitivity of the best performing model (seen with LR and DNN) with NGAL in combination with NT-pro-BNP was 92% and 91% with an AUC of 92, respectively. Standard deviations are shown as error bars.

**Figure 3:**
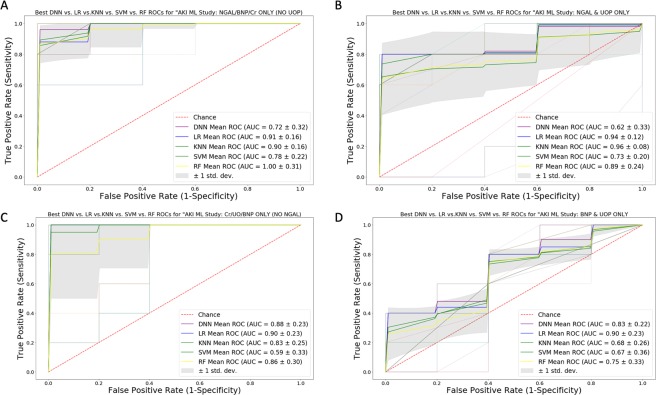
ROC Curve Analysis for Optimized DNN, LR, SVM, *k*-NN, and RF Models with NGAL and/or NT-proBNP: The Figure compares the ROC curves for the best performing models within each AI/ML technique with differing combinations that include NT-proBNP and/or NGAL. False positive rate (1 – specificity) and true positive rates (sensitivity) are reported on the x- and y-axis respectively. Panel A is for NGAL, NT-proBNP, plasma creatinine only. Panel B is for NGAL and UOP only. Panel C is for plasma creatinine, UOP, and NT-proBNP only. Panel D is for NT-proBNP, and UOP only.

**Figure 4:**
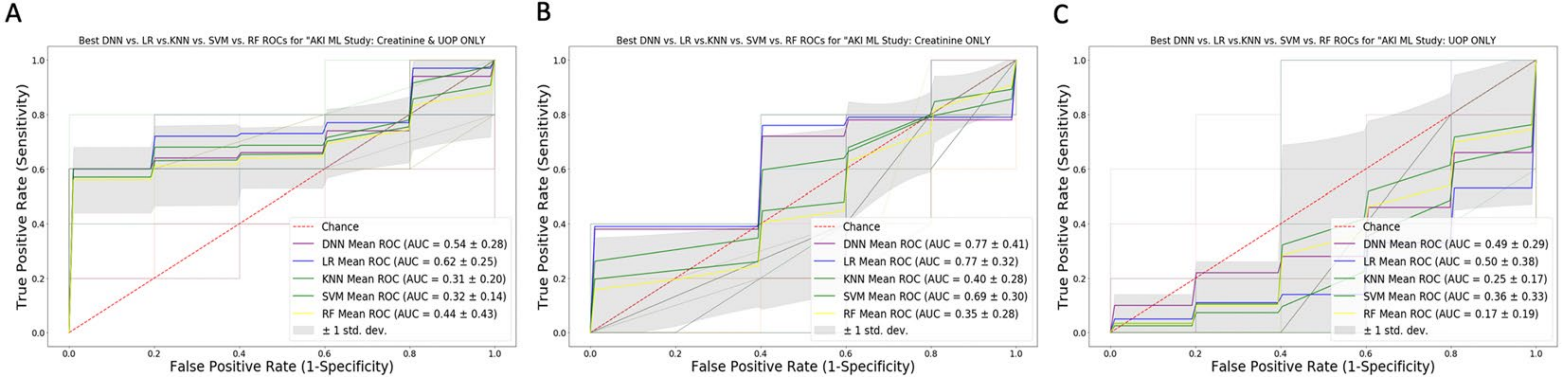
ROC Curve Analysis for Optimized DNN, LR, k-NN, SVM, and RF Models with Traditional AKI Biomarkers: The Figure compares the average ROC curves for the best performing models within each ML method with differing combinations that include UOP and/or creatinine. False positive rate (1 – specificity) and true positive rates (sensitivity) are reported on the x- and y-axis respectively. Panel A is for plasma creatinine and UOP only. Panel B is for plasma creatinine only, and Panel C shows UOP only. Area under the ROC curve values are reported in the bottom right of each Panel.

Table 2 summarizes the mean accuracy for the AI/ML models during the initial validation phase using Cohort A. For the generalization phase (Cohort B), Fig. 2 illustrates the mean accuracy for each biomarker combination using each AI/ML technique. Models using NGAL and NT-proBNP only reported the highest accuracy of 92% and AUC of 0.92 using either DNN or *LR*. The generalization accuracy and AUC of our NGAL and creatinine only model (90% and 91%) was noted within our LR model. Excluding NGAL and retaining the other biomarkers markedly reduced the predictive performance in all 5 of our ML platforms, DNN, LR, k-NN, SVM and RF (generalization accuracy of 55%, 49%, 55%, 41%, 22% and AUC of 71%, 68%, 68%, 63%, 50%, respectively). Notably, in the absence of NGAL, the highest generalization prediction accuracy and AUC was noted within our RF model using creatinine and UOP only (71% and 75%, respectively) and within our DNN model using the combination of creatinine, UOP, and NT-proBNP (55% and 71%, respectively). Figure 3 compares average area under the ROC curve ML model within each method with the best average accuracy for various biomarkers combinations including NGAL and/or NT-proBNP. In contrast, Fig. 4 shows ROC curves for each ML method using traditional AKI biomarkers and excluding NGAL and NT-proBNP.

**Table 2:** Mean Accuracy Using Train/Validation Dataset (Cohort A).

Mean (SD) Accuracy (%) Biomarker Combination	DNN	LR	k-NN	SVM	RF
NGAL, NT-proBNP, UOP, Creatinine	100 (0)	95 (10)	95 (10)	98 (8)	90 (17)
NGAL, UOP, NT-proBNP	88 (17)	88 (17)	90 (17)	83 (23)	90 (12)
NGAL, UOP, Creatinine	100 (0)	98 (8)	98 (8)	98 (8)	93 (16)
NGAL, NT-proBNP, Creatinine	98 (8)	95 (10)	95 (10)	95 (10)	93 (11)
NT-proBNP, Creatinine, UOP	90 (17)	88 (17)	93 (16)	93 (16)	93 (11)
NGAL, NT-proBNP	93 (11)	93 (11)	93 (11)	90 (17)	90 (17)
NGAL, Creatinine	95 (10)	95 (10)	95 (10)	95 (10)	93 (16)
NGAL, UOP	90 (17)	83 (22)	90 (17)	88 (17)	90 (17)
NT-proBNP, Creatinine	90 (12)	88 (13)	88 (13)	90 (12)	90 (12)
NT-proBNP, UOP	85 (20)	85 (20)	78 (21)	85 (20)	90 (12)
Creatinine, UOP	65 (20)	48 (18)	65 (20)	60 (20)	60 (23)
NGAL	85 (17)	83 (16)	85 (17)	85 (17)	85 (17)
Creatinine	68 (16)	58 (39)	65 (32)	68 (20)	65 (20)
UOP	58 (16)	30 (19)	48 (13)	43 (20)	50 (25)

Note: The number of neighbors for k-NN ranted from 1 to 30 for the grid search process on both uniform and distance weight measures. An optimal k-value of 14 was identified within the Minkowski Metric. For RF, 1350 models were generated through the grid search process with multiple random hyperparameter settings. The best performing RF model was comprised of 100 trees (n-estimator = 100) with a maximum depth 3.

Abbreviations: DNN, deep neural network; k-NN, k-nearest neighbor; LR, logistic regression; NGAL, neutrophil gelatinase associated lipocalin; NT-proBNP; N-terminal pro-B-type-natriuretic peptide; RF, random forest; SVM, support vector machine; and UOP, urine output.

## Discussion

This study evaluates the generalizability of a burn population derived ML algorithm for predicting AKI in a mixed burn and non-burned trauma population. Overall, ML is clearly able to provide unique advantages in the context of AKI including the potential to be highly automated via electronic medical record systems, and as observed in previous and current studies, enable early classification of subtle changes for predicting AKI^24–27^. Kate *et al*. used LR, SVM, decision trees, and naïve Bayes to detect undiagnosed AKI in a large population of hospitalized elderly (age >60 years) patients^25^. The study reported area under the ROC curves ranging from 0.66 to 0.74. More recent studies compared the performance of ML versus physician prediction of AKI based on KDIGO criteria to achieve area under the ROC curves of 0.75 and 0.80 respectively for data presented at ICU admission^27^. Optimal performance was achieved with data after 24 hours with area under the ROC curve of 0.89 and 0.95 respectively. The study also suggested ML outperformed NGAL, but did not include NGAL in the model despite its reported benefit^28^.

Our study is unique in that it evaluates ML in a high-risk burn patients and incorporates (rather than comparing) NGAL into the predictive model. Moreover, five ML methods with unique hyperparameter combinations were used in our study to determine which model provides optimal accuracy across the burn-trauma population and generalized to a mixed burn versus non-burned population of varying disease severity.

As predicted, NGAL was found to be predictive of AKI in both burn and trauma surgery populations, even without using ML. The use of NGAL remains highly relevant in this paper since it is presently used in Europe and is expected to become available in the United States for clinical use in the near future^14^. Higher baseline NGAL levels found in our burn patients may be due to their underlying systemic inflammatory response to their injury. Inclusion of natriuretic peptide testing (*i.e*., NT-proBNP) with NGAL and other biomarkers aided in the evaluation of AKI by leveraging the cardiorenal axis^6,7,14^. Notably, NT-proBNP values were higher in both AKI and non-AKI burn patients in Cohort B. Previous studies have shown natriuretic peptides to be useful for predicting over-resuscitation. For our study, mean NT-proBNP values were higher on Cohort B burned patients due to having more severe complications associated with fluid overload. In contrast to NGAL, UOP has been shown previously to perform poorly for AKI especially in burn critical care^6,7,27,29^. The same holds true for plasma creatinine which exhibits high biological variability and less than ideal inter-assay imprecision^30,31^. In our study, although median creatinine and UOP were significantly different, they were clinically similar based on established acceptable values. Creatinine reference intervals at our institution ranges from 0.60 to 1.30 mg/dL, while output targets a range of 0.5 mL/kg/hr^17^ which suggests a range of >30 mL hour in most patients.

Our study highlights the potential power of ML in enhancing the performance of AKI biomarkers in a high-risk population and emphasizes the profound importance of conducting generalization studies across different models. Specifically, our data showed ML was able to enhance the predictive capability and clinical sensitivity of NGAL when it is used in combination with other known biomarkers (*e.g*., NT-proBNP or creatinine). The generalization performance measures of NGAL alone was not surprisingly high with a 92% generalization accuracy, 73% sensitivity, 97% specificity and 85% AUC in 4 out of our 5 ML platforms (DNN, LR, SVM and RF). However, our DNN and LR models provided the best generalization accuracy, sensitivity, specificity and AUC using NGAL with NT-proBNP—achieving an accuracy of 92%, sensitivity of 91%, specificity of 93% and an AUC of 92%. Similar performance was also noted using NGAL with creatinine in our LR model which provided an accuracy of 90%, sensitivity of 91%, specificity of 90% and an AUC of 91%. Performance of *k*-NN using the same biomarker combination (NGAL and creatinine) achieved slightly lower performance measures (84% accuracy, 91% sensitivity, 82% specificity and 87% AUC).

Differences in ML model performance must also be noted between Cohorts A and B. For any ML model, there is a fine balance between over- versus under-fitting data. Extremes in any direction results increases in error rate and bias^32^. In particular, DNN outperform all other models based on Cohort A (Table 2), but did not achieve the same advantages when tested in Cohort B (Fig. 2). This observation could suggest over-fitting likely played a role, however, equally important, Cohort B contained very different non-burned trauma patients which could also reduce the overall performance of DNN. Ultimately, this highlights the importance of evaluating ML model performance with secondary datasets to assess for the degree of fitting and overall generalizability.

In summary, both ML models required the inclusion of NGAL which is expected to become available in the United States. The model with the best generalization accuracy without NGAL showed lower performance measures compared to models that included NGAL as a parameter. Specifically, this was a RF model that relied on a combination of creatinine and UOP only showing an accuracy of 71%, sensitivity of 82%, specificity of 68% and an AUC of 75%. Thus, NGAL may be a transformative biomarker for AKI prediction. Recent studies using ML have not included NGAL or similar biomarkers^24–27^. Interestingly, our ML models perform better than these studies which is likely explained by the inclusion of NGAL with a combination of our algorithms tested (in addition to our DNN model).

Although *k*-NN was not found to be the most generalizable model within this study, the performance of the *k*-NN model in Cohort B was similar to previous burn-focused studies reported in literature based on Cohort A^14^. Interestingly, NT-proBNP alone or in combination with only creatinine and/or UOP deteriorated the accuracy within our DNN, LR, *k*-NN, SVM, ad RF models. However, the addition of NT-proBNP to NGAL lead to our most generalizable models (DNN and LR) which suggests that both of these markers should be included to maintain the optimal predictive performance.

**Figure 5:**
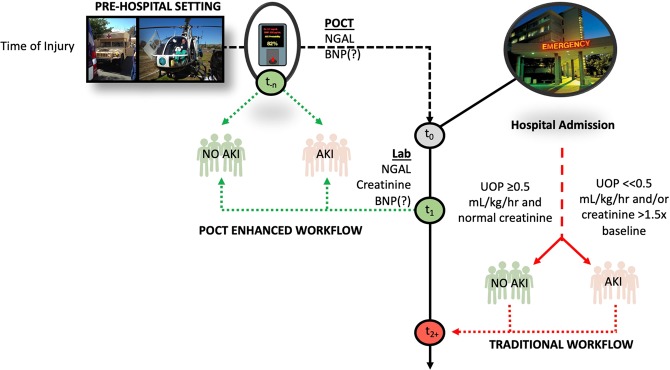
Potential Role of AI/ML for AKI Prediction in Pre-Hospital Setting: Combining point-of-care (POC) testing with AI/ML could be used to enhance diagnostic power in pre-hospital settings. The figure illustrates a conceptual diagram where POC creatinine and NT-proBNP testing is used at a pre-hospital admission time (t_−n_) point and augmented by AI/ML (green pathways). Point-of-care testing data may be then transmitted to an AI/ML algorithm to predict AKI prior to hospital admission. Alternately, AI/ML may also be employed as early as the first day of admission denoted as t_1_. In contrast, traditional workflows (red pathways) relying on urine output and creatinine delay recognition of AKI.

In addition to the above performance enhancements, our ML algorithms predicted AKI an average of 61.8 (32.5) hours (2.5 days) before patients met KDIGO criteria. The potential implications of this finding suggest AI/ML could be also considered for use in pre-hospital settings (*i.e*., ambulance, combat casualty evacuations) to augment point-of-care testing especially when NGAL becomes available in the United States (Fig. 5)^33^.

Limitations of the study include having a modest sample size for the two cohorts and not evaluating the role for ML for AKI occurring beyond the first week of ICU admission. The focus on the first week of ICU stay served to normalize patients in both cohorts to this time period, and also evaluate the role of ML for predicting early AKI.

## Conclusion

Accurate prediction of AKI in a mixed burn/trauma population is feasible using an ML algorithm originally trained for burn patients. This finding highlights the generalizability of ML between these two populations for AKI. Both DNN and *LR*, in particular, provide robust means to predict AKI using both common and esoteric biomarkers of cardiorenal dysfunction. The use of NGAL as a novel biomarker of AKI is further enhanced by ML and should be included in algorithms where feasible. Future studies are needed to evaluate the clinical utility of the ML AKI algorithm in the pre-hospital setting and its impact when used as part of clinical decision support.
